# Is solvent-based dissolution and precipitation an effective substrate pretreatment for the enzymatic depolymerisation of poly(ethylene terephthalate)?

**DOI:** 10.1039/d5fd00061k

**Published:** 2025-08-01

**Authors:** Brooke Wain, Gustavo P. Borin, Elaine M. Rudge, Benjamin Moore, Bruce R. Lichtenstein, Andrew R. Pickford, Victoria L. Bemmer

**Affiliations:** a Centre for Enzyme Innovation, School of the Environment and Life Sciences, University of Portsmouth PO1 2DT UK

## Abstract

Plastics are ubiquitous in modern society; however, their disposal at end-of-life remains challenging. Enzymatic recycling offers a potential low-energy solution to recycling poly(ethylene terephthalate) (PET); however, high-crystallinity substrates such as polyester textiles are recalcitrant to enzymatic hydrolysis. Current amorphisation pretreatments yield substrates amenable to enzymatic digestion; however, they account for a significant percentage of all process electricity requirements. Here we investigate dissolution–reprecipitation with the green solvents gamma-valerolactone and 2-isopropylphenol as a lower-energy pretreatment regime. We find that whilst there is only a minimal decrease in substrate crystallinity, activity of the benchmark PET hydrolase LCC^ICCG^ is increased on all solvent-treated substrates. We show that GVL negatively impacts the thermostability of LCC^ICCG^, and both solvents dramatically decrease enzyme activity, from concentrations as low as 4%, highlighting the need for effective solvent removal following pretreatment. Finally, we show that IPP and GVL are effective for the removal of synthetic dyes from polyester textiles, enabling new applications for these solvents in PET recycling.

## Abstract 2

A dissolution reprecipitation method is presented as an alternative pretreatment for enzymatic recycling of waste PET and shown to enhance the initial rate of reaction, despite a minimal change in substrate crystallinity.

## Graphical abstract

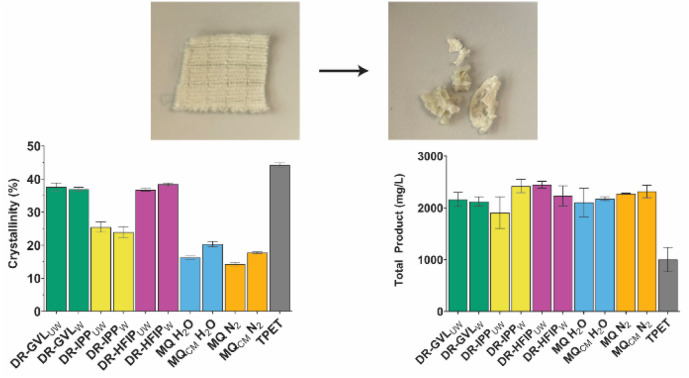

## Introduction

Poly(ethylene terephthalate) (PET) is one of the most widely produced plastics globally, with a broad range of applications, including food packaging, beverage bottles, and textiles. However, its disposal at end-of-life remains a significant environmental challenge, with only around 18.5% of PET produced in the U.S., for instance, currently being recycled.^1^ Whilst recycling of packaging PET is common, it is predominantly done *via* mechanical methods, which can lead to a reduction in the properties of the resulting material.^2,3^ This reduction in the material properties leads to ‘down-cycling’ from packaging to textiles and eventually to carpet, which are destined for landfill at end-of-life.^4^ Enzymatic PET depolymerisation offers an alternative recycling approach, in which ester bonds in the polymer backbone are hydrolysed, yielding the key intermediates bis(hydroxyethyl)terephthalate (BHET) and mono(hydroxyethyl)terephthalate (MHET), which are further broken down into terephthalic acid (TPA) and ethylene glycol (EG) under environmentally benign conditions.^5,6^ These monomers can then be re-polymerised to produce polymers that retain the desirable “virgin-like” mechanical properties.

Despite the promising potential of enzymatic PET recycling, challenges persist, particularly with highly (>30%) crystalline samples, such as polyester textiles. It is believed that the ordered, tightly packed structure of crystalline PET limits enzymatic access, whereas the disordered, flexible structure of amorphous polymers is more readily broken down by enzymes. This relationship, where substrate crystallinity directly impacts hydrolysis efficiency, has been demonstrated in several studies.^7–9^ Two strategies can address this challenge: discovering^10^ or engineering enzymes to tolerate highly crystalline substrates,^11^ and developing lower-energy pretreatments to amorphise the substrate. Current pretreatments involve extrusion, where the plastic is heated above its melting point and then rapidly quenched to trap the material in an amorphous state.^1,12,13^

A recent life-cycle assessment (LCA) highlighted that this pretreatment step accounted for over 90% of electricity requirements for the entire recycling process,^14^ highlighting the need for more efficient approaches. An alternative approach is the use of solvents to dissolve the PET, followed by the rapid addition of an antisolvent to cause the PET to precipitate out of solution.^15–18^ This dissolution–reprecipitation (DR) process has shown promise; however, there are concerns around the use of harsh solvents like hexafluoroisopropanol (HFIP) and their environmental impact. A recent study by Chen *et al.* demonstrated that gamma-valerolactone (GVL), a biomass-derived green solvent, can serve as an effective alternative solvent for the DR process, with water acting as the corresponding antisolvent.^19,20^ This solvent/antisolvent system addresses the environmental concerns associated with other solvent systems; however, its impact on the substrate crystallinity and viability as an enzymatic substrate is unknown. Similarly, 2-isopropylphenol (IPP) is a green lignin-derived solvent^21,22^ which is likely to be able to dissolve PET given its phenolic nature.

In this study, we investigated the impact of the DR method on PET substrate crystallinity with the following three solvents: the commonly used HFIP, and the two ‘green’ solvents GVL and IPP. We also analysed the solvents’ impact on enzymatic activity, specifically the tolerance of the well-characterised PET-degrading enzyme LCC^ICCG^, a variant of the cutinase enzyme leaf and branch compost cutinase (LCC), originally identified from a soil metagenomic screen.^12,23,24^ This variant has been shown to exhibit enhanced activity, with PET hydrolysis approaching 100% depolymerisation in 96 hours, making it one of the most promising candidates for enzymatic recycling applications. In addition, we evaluated the feasibility of solvents as a method of extracting potentially valuable dye molecules from textile substrates.

## Results and discussion

### Dissolution–reprecipitation of PET substrates

**Fig. 1:**
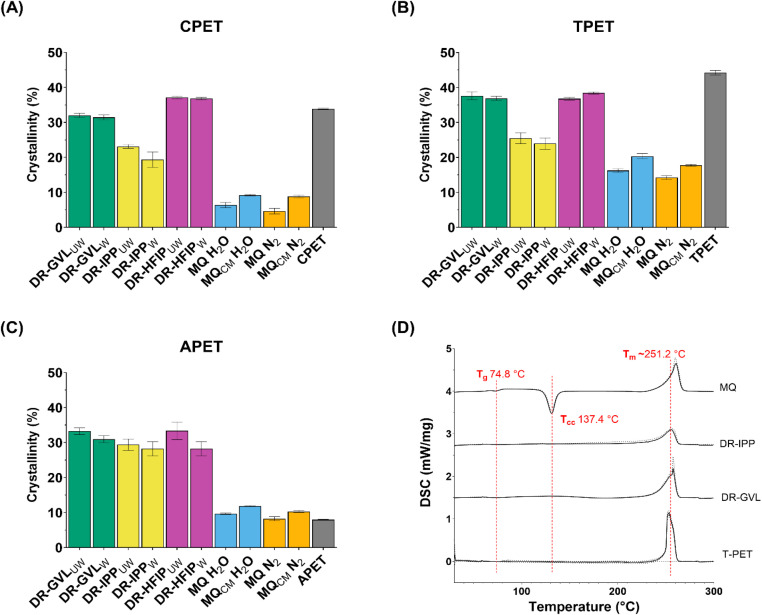
Crystallinity (*X*_C_) of PET substrates subjected to various pretreatments. (A–C) Bar charts showing the percentage *X*_c,_ with lower *X*_c_ values indicating increased amorphisation, determined by Differential Scanning Calorimetry (DSC), with standard deviation shown as error bars of (A) CPET, (B) TPET and (C) APET, after solvent (GVL, IPP, and HFIP) treatment *via* dissolution–reprecipitation (DR), washing, melt quenching (MQ) in liquid nitrogen (LN_2_) or water (H_2_O), and milling (CM) to 0.25 mm. Washed (W) and unwashed (UW) treated PET substrates are also indicated. (D) Example DSC chromatograms of TPET subjected to DR with GVL and IPP and MQ with N_2_. The glass transition temperature (*T*_g_) is observed at approximately 75 °C, and the melting temperature (*T*_m_) of PET is observed around 250 °C. For the MQ amorphised sample, the cold crystallisation temperature (*T*_cc_) at approximately 137 °C is evident. The *T*_g_, *T*_cc_, and *T*_m_ are highlighted by labelled red dashed lines.

The two high-crystallinity PET substrates, semi-crystalline PET powder (CPET, 33.8 ± 0.2% crys.) and polyester textile (TPET 44.2 ± 0.5% crys.), along with an amorphous PET powder (APET 7.9 ± 0.1% crys.) control, were subjected to solvent treatment using the DR process with GVL, HFIP and IPP. A melt-quench (MQ) extrusion-like pretreatment, quenched in liquid nitrogen (N_2_), was included as a control, based on its previously described effectiveness in achieving amorphisation.^25^ After treatment, the polymer samples were washed and analysed before and after treatment using Differential Scanning Calorimetry (DSC), as shown in Fig. 1.

Both CPET and TPET exhibited similar patterns of crystallinity (*X*_C_) after either solvent or MQ treatments (Fig. 1A and B). Initially, both substrates had high *X*_C,_ which was reduced slightly by GVL treatment (CPET reduced to 31.4% crystallinity, TPET to 36.9%), but more notably after treatment with IPP (19.3% for CPET, 23.9% for TPET). A post-treatment wash with water had minimal impact on the *X*_C_ values, suggesting that the solvent treatment was mainly responsible for the changes in PET structure. Where there were samples (*e.g.* HFIP-treated TPET) that exhibited slight increases in *X*_c_ after washing, this could be attributed to the incomplete solvent removal or evaporation, which might lead to the reorganisation of crystalline regions as previously suggested.^26^ These findings support the idea that solvent treatment can effectively reduce the crystallinity of PET, though not to the extent observed with MQ treatment. The effectiveness of IPP in promoting amorphisation is likely due to it being a better solvent for PET, which dissolved much more readily in IPP than GVL, and can more easily disrupt the crystalline structure and facilitate reorganisation into an amorphous state, in good agreement with results previously seen by others.^15,27^

Interestingly, the effect of HFIP on crystallinity was not consistent with that of the other solvents, as it showed an increase in *X*_c_ for both CPET and TPET (Fig. 1A and B). This finding contrasts with previous studies where polar aprotic solvents were used for PET solubilisation, suggesting that HFIP would effectively dissolve and amorphise PET.^28–32^ This difference may be attributed to the experimental method used where due to HFIP’s unique solvent properties, such as low boiling point (Table 1), we employed a sonicator to aid in dissolving the PET, rather than heating it in a round-bottom flask with stirring, which has previously been shown to accelerate crystallisation through localised nucleation;^33^ however, it is important to note that sonication was not used during the precipitation process.

**Table 1:** Physiochemical properties of solvents^34–36^

Solvent	Formula	log *P*^a^ value	Boiling point (°C)	Flash point (°C)	Miscible in water?	Anti-solvent	Risk
GVL	C_5_H_8_O_2_	−0.27	207	96	Yes	Water	H315, H319
IPP	(CH_3_)_2_CHC_6_H_4_OH	2.88	212	104	No	Ethanol	H302, H314, H318
HFIP	C_3_H_2_F_6_O	−1.38	58.2	>100	Yes	Water	H314, H361fd, H373
DMSO	(CH_3_)_2_SO	−1.35	189	203	Yes	—	—
MeCN	C_2_H_3_N	−0.34	82	2	Yes	—	

a log_10_ of the partition coefficient (*P*); indicative of solubility of a chemical according to its oil-to-aqueous phase. log *P* < 1: more solubility in water; log *P* > 1: lesser solubility in water. log *P* H_2_O: −1.38.

The most noticeable amorphisation was observed in the MQ-treated substrates (Fig. 1). For all samples, crystallinity was reduced by the MQ process to a range generally better tolerated by PET hydrolases (*X*_c_ < 20%).^5,9,25^ The lack of significant difference (*p* = 0.06, one-way ANOVA) in the resultant *X*_c_ between quenching in H_2_O or LN_2_ suggests that the cooling rate, rather than the medium, is key in preventing re-crystallisation. Interestingly, while milling has been shown to reduce crystallinity,^37^ milling of MQ-treated samples increased crystallinity. One possible explanation is that the MQ-treated sample, trapped in a high-energy disordered state, underwent reorganisation into a more crystalline state during milling, enabled by input of mechanical energy, as seen previously where *X*_C_ increased with increasing milling time for PET with low initial crystallinity.^38,39^ All solvent- and melt-quench-treated samples showed a significant difference in crystallinity when compared to untreated samples (*p* < 0.05, one-way ANOVA test) except when comparing solvent-treated TPET samples.

### Assessing enzyme activity against solvent-treated PET

**Fig. 2:**
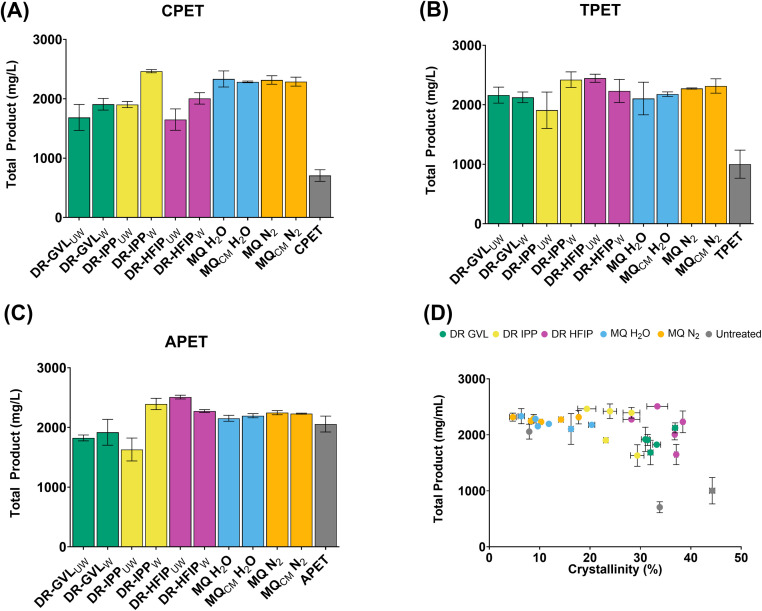
Activity of LCCICCG against pre-treated (A) semi-crystalline PET powder (CPET), (B) polyester textile (TPET,) and (C) amorphous PET powder (APET,) substrates. Reactions were carried out in triplicate, with 500 μL of 0.5 M potassium phosphate buffer (pH 8.0) containing 500 nM LCCICCG, at 65 °C for 96 hours with continuous agitation at 300 rpm. Enzyme-free reactions served as negative controls. Degradation products from PET hydrolysis were quantified by HPLC, measuring the release of the monomers TPA, MHET, and BHET. Data are presented as mean ± standard deviation (SD) of triplicate reactions, UW: unwashed; W: washed; CM: cryo-milling. The relationship between crystallinity and total product yield is shown in panel D, and a weak negative correlation is observed (corr = −0.5, R squared = 0.27).

The pretreated CPET, TPET, and APET samples were incubated with LCC^ICCG^ for 96 hours to analyse the impact of the pretreatments on enzyme activity. The total product yield following enzymatic PET hydrolysis is shown in Fig. 2. As expected, for the untreated samples the extent of depolymerisation generally showed an inverse dependency on substrate crystallinity, in keeping with previously observed results.^7–9^ Interestingly, it is apparent that solvent treatment enhances enzyme activity for substrates with high initial crystallinity (CPET and TPET), despite no substantial reduction in the overall crystallinity being observed and only a weak negative correlation between crystallinity and extent of depolymerisation (Fig. 2D). This contrasts with previous studies, which have suggested the activity of LCC^ICCG^ on PET is essentially capped on samples around 22–25% crystallinity.^40^ Visual inspection of the solvent-treated PET samples showed changes in morphology based on the solvent used. HFIP-precipitated PET appeared more solid but had a porous, sponge-like texture, whereas PET precipitated with GVL and IPP appeared flakier. This change in morphology may suggest that residual solvents and water are having a plasticising effect on the PET, making the intracrystalline polymer chains more flexible and aiding enzyme access. An alternative explanation is the change in morphology has increased the surface area available to the enzyme for binding and hydrolysis; however, this has not been confirmed and would require further study.

Of interest is the effect of washing on solvent-treated samples. In the case of GVL and IPP, washing the substrates resulted in a slight increase in enzymatic activity (Fig. 2), suggesting that residual solvent might be inhibiting the enzyme activity in unwashed samples. It is known that organic solvents, such as GVL and IPP, can interfere with enzyme–substrate interactions in several ways, either by disrupting the tertiary structure and impacting thermal stability and/or the active site, or affecting the enzyme’s ability to bind effectively to the substrate by altering the surface properties of the PET polymer.^41^ In contrast, the effect of washing was not seen for HFIP-treated samples, most likely due to the boiling point of HFIP (58.2 °C) being below the depolymerisation temperature of 65 °C, causing any residual HFIP to evaporate.

### Impact of organic solvents on the thermal stability and activity of LCC^ICCG^

To understand the cause of the apparent enzyme inhibition by GVL and IPP, we decided to investigate the impact of the solvents on the apparent melting temperature (*T*_m_) of LCC^ICCG^ by DSC. Of the solvents used for polymer pretreatment, only GVL was progressed for these analyses, owing to the poor compatibility of IPP and HFIP with the instrumentation due to solvent immiscibility and volatility (Table 1). In addition, we studied the effect of dimethyl sulfoxide (DMSO), a solvent well tolerated across multiple enzyme classes including PETases,^10^ and acetonitrile (MeCN), which is known to disrupt protein structure, particularly at higher concentrations.^42^

**Fig. 3:**
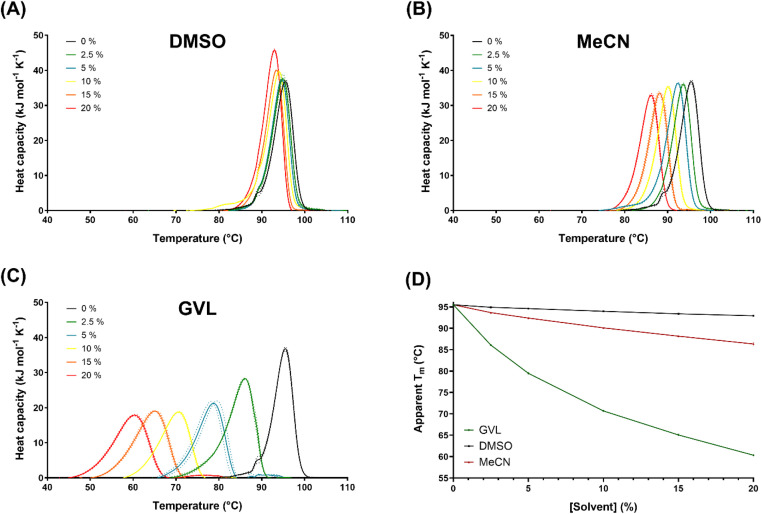
Tolerance of LCC^ICCG^ to varying concentrations of DMSO, acetonitrile (MeCN) and gamma-valerolactone (GVL) assessed by differential scanning calorimetry. (A–C) Thermograms showing the apparent melting temperature (*T*_m_) of LCC^ICCG^ in the presence of increasing solvent concentrations (0%, 2.5%, 5%, 10%, 15% and 20%) of (A) DMSO, (B) MeCN and (C) GVL. Solid lines represent the average of triplicate reactions, and the dotted lines indicate the standard deviation (SD) at each solvent concentration. (D) Comparative plot of the apparent *T*_m_ values of LCC^ICCG^ for DMSO (black), MeCN (red) and GVL (green). Data points represent mean values, with error bars showing SD.

At a scan rate of 1.5 °C min^−1^, LCC^ICCG^ exhibited an apparent *T*_m_ of 95.5 °C in the absence of solvent, consistent with previous findings by Tournier *et al.*^24^ When increasing the concentration of DMSO from 0 to 20% (v/v), there was little change in the resulting thermograms, with a very slight decrease in the apparent *T*_m_ from 95.5 °C to 92.9 °C (Fig. 3A). Conversely, LCC^ICCG^ was destabilised by the presence of MeCN, with the apparent *T*_m_ decreasing to 86.3 °C at 20% (v/v) MeCN (Fig. 3B). The impact of GVL on the thermostability of LCC^ICCG^ was most notable, with thermal unfolding occurring at much lower temperatures compared to the other solvents (Fig. 3C). At only 2.5% (v/v) GVL, the apparent *T*_m_ decreased to 86.1 °C, lower than that observed at the highest concentration of either DMSO or MeCN. Further increases in GVL concentration caused a dramatic reduction in the apparent *T*_m_, dropping to 60.3 °C at 20% GVL, suggesting a greater susceptibility to thermally-induced denaturation at higher solvent concentrations. At high solvent loadings, pH optima are likely to be affected by organic solvent content, but to ease comparison these effects were not considered.

One possible explanation for this increased destabilisation could be the lower solubility of GVL in aqueous environments compared to DMSO and MeCN, as reflected by its lower partition coefficient (log *P*) value (Table 1), with interactions with GVL stabilising unfolded intermediates presenting hydrophobic core residues to the solvent. The effect would be a decrease in thermostability and activity of the enzyme.

**Fig. 4:**
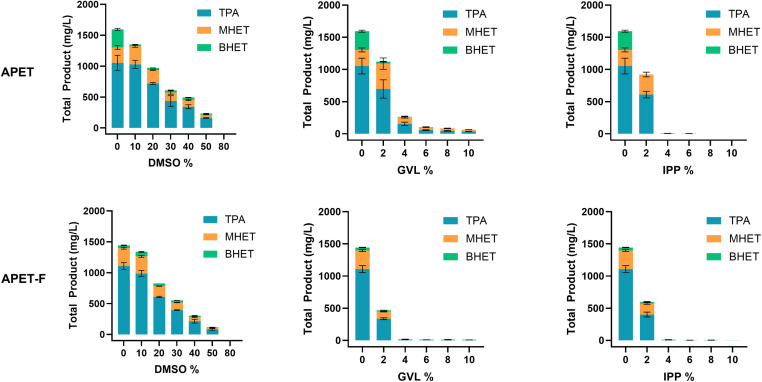
LCC_ICCG_ tolerance to organic solvents dimethyl sulfoxide (DMSO), gamma-valerolactone (GVL), and 2-isopropylphenol (IPP) at varying concentrations on an amorphous PET film (APET-F) and powder (APET). Enzyme activity was assessed using two known enzymatic hydrolysable substrates, APET and APET-F. Solvent concentrations ranged from 0% to 100% in 10% increments; however, due to a marked reduction in activity at higher concentrations of GVL and IPP, detailed analysis was focused on the 0% to 10% range in 2% increments for these solvents. Total degradation products released from PET hydrolysis were quantified by high-performance liquid chromatography (HPLC). Monomers TPA, MHET, and BHET released are shown as mean values with standard deviation (SD) from replicate experiments, with TPA in blue, MHET in orange, and BHET in green.

With the apparent strong impact of GVL on enzyme thermostability, we decided to investigate how this reduction in apparent *T*_m_ impacted overall activity. For this screening, we used GVL and IPP from the initial pretreatment trials, and DMSO as a control as it was shown to have minimal impact on the apparent *T*_m_ (Fig. 4). Amorphous PET, in powdered (APET) and film (APET-F) forms, was selected as a substrate due to its high susceptibility for enzymatic hydrolysis (Fig. 2C). HFIP was excluded from this screen as its boiling point of 58.2 °C is below the optimal temperature for LCC^ICCG^ activity at 65 °C.

As anticipated, LCC^ICCG^ demonstrated greater tolerance to DMSO, retaining over 88% of its activity against both APET (Fig. 4A–C) and APET-F (Fig. 4D–F) at 10% DMSO. In contrast, its activity was almost completely inhibited at 4% solvent concentration for both GVL and IPP. Interestingly, despite DMSO having minimal impact on the thermostability of LCC^ICCG^ (Fig. 3A and D), enzyme inhibition was observed at higher solvent concentrations, suggesting an alternative inhibition mechanism, such as an interaction at the active or binding sites of the enzyme. Solvent properties play a role in determining the extent of interference and modulating enzyme performance – the reaction system with IPP is heterogeneous which may encourage partitioning of PET from the water-dissolved enzyme or support denaturation of the enzyme at the phase barrier, features not observed with GVL.

In addition to solvent dependence, enzyme inhibition was found to be substrate dependent in the presence of GVL, with a stronger inhibitory effect seen on APET-F compared to APET. This phenomenon has been observed in several PET hydrolases and is often attributed to differences in electrostatic interactions^43,44^ or protein dynamics.^44^

### Solvents for dye extraction

**Fig. 5:**
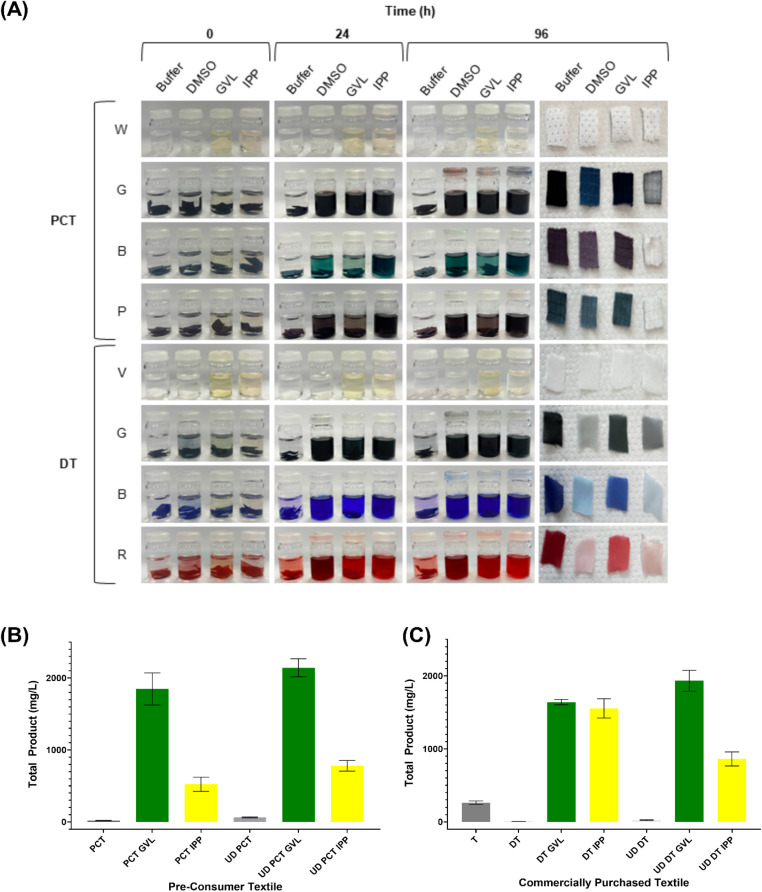
Textile dye extraction using solvents. (A) 96-hour solvent incubation. Pre-consumer commercial textiles (PCT) in four colours (white (W), graphite (G), blue (B) and purple (P)), and virgin commercial TPET (V) purchased from Whitely, dyed using Rit synthetic dye in three colours (graphite (G), sapphire blue (B) and racing red (R)) were analysed. All textiles were incubated with 5 mL of DMSO, GVL, IPP and 0.1 M potassium phosphate buffer as a control. Reactions were left for 96 hours, with photos taken at 0, 24 and 96 hours. After incubation, textile samples were removed, washed with 100% ethanol, then with water and dried at 50 °C overnight. Enzymatic breakdown of textile samples following dissolution and reprecipitation (DR) with GVL and IPP with (UD) and without dye removed. Reactions conducted in 500 μL of 0.5 M potassium phosphate buffer (pH 8.0) containing 500 nM LCC^ICCG^, at 65 °C for 96 hours with continuous agitation at 300 rpm with (B) PCT G and (C) DT G textile samples. Total degradation products released from PET hydrolysis quantified by high-performance liquid chromatography (HPLC), mean taken from triplicate reactions and standard deviation plotted as error bars. Virgin Whaleys undyed voile textile (TPET) used as an additional control for DT.

Given the impact of these solvents on protein stability and activity, we explored an alternative application: their potential for dye extraction from textiles, for recycling of valuable molecules and removal of potential environmental pollutants. We tested both commercially purchased 100% polyester dyed pre-consumer textiles (PCT) and laboratory-manually dyed virgin white Whaley’s Voile TPET (DT), each textile set being stained with three different dyes. These textiles were incubated with 0.1 M potassium phosphate buffer, DMSO, GVL and IPP to assess whether the solvents could effectively extract dye from the materials. Images of the textile reaction vials were captured before and after a 96-hour reaction period and following subsequent drying (Fig. 5).

After the 96-hour incubation, it was clear that all three solvents were effective in extracting dye from both the PCT and DT samples, as shown in Fig. 5A. The control samples showed no notable dye removal.

Dye extraction after 24 hours suggested that IPP extracted the dye more rapidly than DMSO, with GVL showing the slowest rate of extraction, as reflected in the colour of the solvent solutions. This trend is most likely attributed to the solvents’ different polarities and their ability to interact with and solvate the dye molecules, with IPP being the most effective due to its higher solvating power for a wider range of dye types. This was corroborated by the final images of the dried textile substrates in Fig. 5A. The observed dye extraction pattern in the vials matched the changes seen in the dried textiles, with the same relative extraction efficiencies for both the PCT and DT substrates.

Greater dye removal in the lab-dyed samples was also observed and is likely due to the dyeing process. In the lab, the dye is added after the fabric is produced, allowing for weaker dye–fibre bonds that are easier to extract. In contrast, commercial textiles have dye added during manufacturing under heat and pressure,^45,46^ forming stronger, more permanent bonds that are harder to break, resulting in less efficient dye removal.

To analyse the impact of dye on the enzymatic activity on the PCT and DT substrates, the graphite samples of PCT and DT were subjected to dye removal, and all samples subjected to DR treatment, Fig. 5B and C, respectively.

For the untreated, dyed and undyed PCT and DT substrates, there is a lack of activity, corroborating previous observations on highly ordered substrates.^7,9^ Although activity was observed for the virgin textile, this was reduced compared to the activity seen for the powder textile (TPET) in Fig. 2B. This increase could be the result of the milling treatment increasing the surface area and subsequent reaction rate.^47^

The DR treatment for all samples, showed an increase in activity with both GVL and IPP, compared to their untreated samples. Except for DT, there is a noticeable increase in activity following treatment with GVL then with IPP. This could be attributed to the incomplete removal of IPP, with its remaining presence interfering and denaturing the protein, corroborating with the data seen in Figure 4C and F, where low levels of IPP have a noticeable impact on activity against amorphous PET substrates. This was not seen for DT DR-treated samples. This does not follow what was expected and could be attributed to complete IPP removal during the EtOH and water washing steps following the DR treatment.

Interestingly, for these two dyed textiles, the impact of dye removal prior to DR treatment did not have a substantial impact on activity, and similar activity was seen for both dyed and undyed samples with their respective DR treatment.

## Conclusions

In this study, we aimed to explore solvent-based amorphisation as a potential lower-energy alternative to traditional MQ pretreatment methods for enzymatic depolymerisation of high-crystallinity (>35%) PET substrates,^8^ such as polyester textiles. Whilst the MQ process is more efficient than solvent-based approaches at amorphising the substrate, solvent dissolution–reprecipitation does appear to enhance enzyme activity on highly crystalline substrates. We suggest that this is due to the solvents plasticising the PET specifically by disrupting the interactions between adjacent polymer chains, especially upon precipitation, making them more accessible for enzymatic hydrolysis.

We investigated enzyme tolerance to various solvent concentrations and found that even low concentrations of GVL and IPP significantly affected enzyme activity, and GVL impaired enzyme thermostability, highlighting the importance of efficient solvent removal following any feedstock pretreatment involving either of these green solvents. Additionally, we demonstrated their potential as effective agents for dye removal from textiles, which could be implemented in a simultaneous substrate pretreatment and dye removal approach, increasing economic viability of the process. This is supported by the analysis of dye removal and DR on enzymatic activity, which proves that dye can be removed and not have an inhibitory effect on the DR pretreatment, which is necessary for activity to be observed when compared to virgin substrates.

Ultimately, while solvent-mediated PET pretreatments show potential, their scalability and efficiency are limited by challenges in solvent removal and enzyme tolerance. The MQ method remains the most optimal choice for achieving high amorphisation and enzymatic activity. Further research and optimisation of any solvent treatments will be essential for advancing sustainable PET recycling technologies, especially for highly crystalline substrates.

## Materials and methods

### PET substrate preparation and micronisation

APET-F (ES30-FM-000145) and CPET powder (ES30-PD-006031) were purchased from Goodfellow. The APET-F sheets (0.25 mm thickness) were punched into stadium-shaped samples (10 mm × 13 mm) for screening experiments. APET powder was produced through micronisation *via* cryo-cutting (CC) at 2400 rpm in a Retsch SM300 cutting mill with a 4 mm sieve and subsequently cryo-milled (CM) at 18 000 rpm in a ZM200 centrifugal mill, with a 0.12 mm ring sieve. All CC and CM substrates were dried overnight at 50 °C to eliminate any residual moisture before further use and the particle size was confirmed using a CAMSIZER X2 (Microtrac MRB) with *X*_C_ measured using DSC.

The TPET polyester textile Voile White was purchased from Whaleys (Bradford) company (England, UK) and was cut into 1 cm × 1 cm squares, washed three times with 70% ethanol, washed three times with water and then dried at 50 °C for 48 hours.

### Melt quench treatment

PET substrates were heated to 280 °C until molten, then rapidly transferred to liquid nitrogen (N_2_) or ice-cold water (H_2_O) for quenching. Substrates were cooled for 5 minutes before being placed in a drying oven at 50 °C overnight. The MQ-treated substrates were then micronised by CC and CM to 0.25 mm and dried, as described above.

### Solvent dissolution and reprecipitation treatment

The DR method was varied according to solvent boiling point. For GVL (Sigma-Aldrich, ≥99.5%) and IPP (Sigma-Aldrich, ≥99%), 6 mL of solvent was added to a sealed 50 mL round-bottom flask containing a small magnetic stir bar. The solvent was heated to 180 °C with agitation at 400 rpm. Once the solvent had equilibrated to the desired temperature, PET (0.6 g) was added to the flask to give 10% solids loading and allowed to dissolve for 30 minutes. Where HFIP (Sigma-Aldrich, ≥99%) was used as a solvent, 6 mL was placed in a 50 mL glass vial with 0.6 g of PET and sonicated at 40 °C for up to 4 hours, until all substrates had dissolved. For all solvents, once dissolution was confirmed visually, the mixture was removed from the heat, and 6 mL of ice-cold antisolvent (water for GVL and HFIP, and 100% (v/v) ethanol for IPP) was quickly added to precipitate the PET material. The flask was immediately transferred to an ice bath to further facilitate precipitation.

All resulting precipitated PET material was filtered, and an unwashed (UW) sample collected. The remaining substrate was washed (W) with 350 mL of antisolvent to remove any residual solvent. Finally, the material was lyophilised to ensure the complete removal of moisture, resulting in a dry, reprecipitated product suitable for further analysis *via* polymer DSC.

### Polymer differential scanning calorimetry

Substrate crystallinity was confirmed *via* DSC analysis using a DSC 214 Polyma (Netzsch). 5–10 mg of PET was placed in an aluminium pan and heated from 25 °C to 300 °C at a rate of 10 °C min^−1^ under a nitrogen atmosphere. The resulting peaks were integrated, and the average area (J g^−1^) was used to calculate substrate *X*_C_ using the equation: % crystallinity (*X*_C_) = (Δ*H*_m_ − Δ*H*_c_)/Δ*H*_m_^0^, where Δ*H*_m_ represents the enthalpy of melting of the sample, Δ*H*_c_ is the enthalpy of crystallisation, and Δ*H*_m_^0^ is the enthalpy of melting for a theoretical 100% crystalline PET sample, with a reference value of 140.1 J g^−1^. Data analysis was performed using Proteus Analysis software.

### Enzyme production

The DNA sequence encoding the protein of interest, LCC^ICCG^, was synthesised by Twist Biosciences and cloned into the pET21b(+) vector, which included a C-terminal hexa-histidine tag, shown in Table 2. The plasmid was optimised for expression in *Escherichia coli* (*E. coli*) BL21 (DE3) Gold cells. For protein expression, a starter culture was initially grown in minimal media^48^ supplemented with kanamycin (50 μg mL^−1^) at 37 °C overnight. The starter culture was then used to inoculate 2 L flasks containing 1 L of autoinduction media at a 1 : 100 dilution, and the culture was grown at 28 °C for 24 hours.

**Table 2:** Expressed amino acid sequence of LCC^ICCG^

Enzyme	Protein sequence^a^
LCC^ICCG^	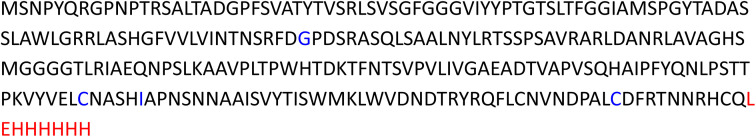

a Sequences colour-coded with red indicating the 6×HIS tag and blue denoting the amino acid mutation residues introduced to LCC^ICCG^ from LCC.

After expression, cells were harvested by centrifugation at 10 000 × *g* for 15 minutes at 4 °C and resuspended in a lysis buffer containing 300 mM NaCl, 10 mM imidazole, 20 mM Tris, pH 8.0. Cell lysis was performed using probe sonication at 40% amplitude for a total on-time of 6 minutes, keeping samples on ice throughout. The lysate was clarified by centrifugation at 55 000 × *g* for 45 minutes at 4 °C and filtered through a 0.45 μm MCE membrane (Fisher Scientific) prior to purification. The clarified supernatant was applied to a 5 mL HisTrap HP Ni-NTA column (Cytiva) pre-equilibrated in binding buffer. Bound protein was eluted using a step gradient of elution buffer (20 mM Tris–HCl, pH 8.0, 300 mM NaCl, 500 mM imidazole), progressing through 3%, 50%, and 100% steps.

Eluted fractions were analysed by SDS-PAGE, and those containing the target protein were pooled and concentrated using a 10 kDa molecular weight cut-off (MWCO) polyethersulfone (PES) centrifugal filter unit (Amicon Ultra). Further purification was performed by size exclusion chromatography (SEC) on a HiLoad Superdex 75 pg 16/60 column (Cytiva) equilibrated with 0.5 M potassium phosphate buffer, pH 8.0. Protein-containing fractions were assessed by SDS-PAGE with Coomassie Blue staining. Fractions containing the target construct of LCC^ICCG^ were pooled and stored for downstream analyses.

### Enzyme activity assays

To assess the success of reducing the *X*_C_ of PET substrates through various pretreatments (DR and MQ), LCC^ICCG^ was incubated with the treated substrates (CPET, TPET and APET), with untreated PET substrates serving as controls. 1.5 mL microtubes containing 10 mg (2% w/v) of PET substrate were incubated with 697 nM LCC^ICCG^ (1 mg enzyme per 1 g substrate). The reactions were performed in triplicate, with a total volume of 500 μL of 0.1 M potassium phosphate buffer, pH 8.0. Reaction tubes were incubated in thermomixers (Eppendorf, Germany) at 300 rpm and 65 °C for 96 hours. Negative controls, containing substrate and buffer without enzyme, were also incubated in duplicate for each condition. Samples were quenched by adding an equal volume of HPLC-grade methanol, then spun at 14 000 rpm for 10 minutes before being diluted to an absorbance of 1 at 240 nm. The quantification of the aromatic monomers TPA, MHET, and BHET was carried out using a 1260 Infinity II LC System (Agilent, USA) equipped with a diode array detector set to 240 nm following the method outlined below.

To assess solvent tolerance, activity of LCC^ICCG^ was measured using the conditions described above, with varying concentrations of DMSO, GVL, and IPP mixed with 0.1 M potassium phosphate buffer. Activity was analysed against the two substrates, APET and APET-F, which typically yield higher monomer yields.

Solvent concentrations were initially tested from 0% to 100% in 10% increments. However, GVL and IPP showed reduced activity at higher concentrations and, subsequently, the analysis was narrowed to the range of 0% to 10% in 2% increments, with GVL being further assessed from 0% to 1% in 0.2% increments to account for its stronger inhibitory effect. All solvent solutions were prepared by mixing the enzyme buffer with the respective organic solvent in 10 mL glass vials, and the pH of each solution was individually adjusted to pH 8.0.

### Monomer quantification by HPLC

HPLC quantification was performed using a method previously published.^49^ Samples were auto-injected at 10 μL onto a pre-equilibrated C18 Kinetex LC column (00B-4605-AN) equipped with a column guard, at a flow rate of 1.1 mL min^−1^, using a solution of 0.1% formic acid and MeCN as the mobile phase. Samples were eluted with an isocratic elution at 13% mobile phase for 0.87 minutes, followed by a step increase to 95% mobile phase for 1.12 minutes, and re-equilibration at 13% mobile phase until a total run time of 3.6 minutes. Between sample sets, MeCN was injected to wash the system and minimise sample contamination. Samples were prepared to a known dilution to achieve an absorbance at 240 nm of approximately 1.0 before being loaded onto the column using an automatic sampler (Agilent). Peaks were integrated using Agilent’s OpenLab software, and product quantification was performed using calibration curves generated from known standards of TPA, MHET and BHET.

### Protein differential scanning calorimetry

Protein stability was assessed *via* protein DSC using a Microcal PEAQ-DSC Automated (Malvern Panalytical) in the presence of varying concentrations of DMSO, ACN and GVL. Solutions of the solvents at concentrations of 0%, 2.5%, 5%, 10%, 15%, and 20% were prepared by mixing 50 mM sodium phosphate buffer at pH 7.5 and the pH of each solution individually adjusted. pH optima are likely to be affected by organic solvent content but to ease comparison these effects were not considered.

The protein was prepared in the corresponding solvent-buffer mixtures to a concentration of 0.5 mg mL^−1^ and for each reaction condition 325 μL was added to a 96-well plate, in triplicate. The temperature was increased from 30 °C to 120 °C at a single scan rate of 1.5 °C min^−1^ using the low feedback mode. The resulting thermograms were analysed using the instrument’s control and analysis software. The data analysis included baseline correction, buffer subtraction, and determination of the apparent melting temperature (*T*_m_). The heat capacity (kJ mol^−1^ K^−1^) *versus* temperature (°C) thermograms were further analysed with CalFitter v2.0 for kinetic modelling of protein denaturation.^50^

### Dye extraction

DyeMore (Rit) dye for synthetics was used in three colours: graphite (G), sapphire blue (B) and racing red (R). Virgin Whaleys TPET was dyed following the manufacturer’s instructions. Briefly, a dye solution was prepared by heating 1 L of water to 93 °C, followed by the addition of 228 mL of dye. The virgin TPET (500 g) was first washed with soap in warm water, then immersed in the dye solution. The mixture was continuously stirred, and the TPET was incubated in the dye for 1 hour. After incubation, the dyed TPET was removed, rinsed with water, and dried at 40 °C overnight. The experiment used the DT variants (G, B and R) and the virgin (V) TPET, along with four 100% polyester PCT coloured white (W), G, B and purple (P). All textile substrates were cut into 1 cm × 1 cm squares, washed three times with 70% ethanol followed by water, and then dried at 40 °C for 48 hours prior to dye extraction.

For the dye extraction process, 5 mL of solvent (GVL, IPP or DMSO) was added to a glass vial, with 0.1 M potassium phosphate buffer (pH 8.0) used as a control. To each solution, 0.5 g (10% w/v) textile substrate was added, the vial sealed with a solvent-compatible lid, and the reaction mixture incubated at 65 °C on a rocker set to 120 rpm. After 96 hours of incubation, the textile samples were removed, washed with water, and dried overnight at 50 °C. Photographs of the dye-extraction vials were taken every 24 hours, as well as of the removed substrates after the 96-hour incubation. The experiment was repeated for PCT-G and DT-G substrates using GVL, IPP, and buffer, with images taken every 2 hours over a 24-hour period.

### Degradation analysis following dye extraction

The graphite-coloured samples of both the in-house DT and PCT were subjected to DR with GVL and IPP following the method outlined above. In 50 mL of IPP, 5 g of substrate samples were incubated for 48 hours at 65 °C on a rocker set to 120 rpm. Following dye removal, the substrate samples were washed with 100% EtOH and water, then dried for 48 hours at 50 °C. The undyed substrates were then subjected to DR with GVL and IPP.

Enzyme activity was analysed following the method outlined above, and across the dyed and undyed, untreated and DR-treated, G-coloured DT and PCT textile samples, along with TPET as a control for DT. Following analysis *via* HPLC, the total product (mg L^−1^) was calculated.

## Author contributions

Amorphisation experiments were performed by BW, GPB, BM, BRL and VLB. Protein production and purification was performed by BW and EMR, DSC measurements and analyses, as well as the dye extraction experiments and enzyme activity screening, were performed by BW and GPB. VLB and ARP conceptualised and supervised the work, and VLB, BRL and ARP acquired funding. BW and VLB wrote the manuscript, which was reviewed by all authors.

## Conflicts of interest

There are no conflicts of interest to declare.

## Data Availability

Data for this article are available at University of Portsmouth Research Portal at https://doi.org/10.17029/ffe479fa-1491-4f26-9dae-78f7e9b5508d.
